# Pathogenesis of recent Lassa virus isolates from lineages II and VII in cynomolgus monkeys

**DOI:** 10.1080/21505594.2022.2060170

**Published:** 2022-04-18

**Authors:** Mathieu Mateo, Jimmy Hortion, Emeline Perthame, Caroline Picard, Stéphanie Reynard, Alexandra Journeaux, Clara Germain, Xavier Carnec, Nicolas Baillet, Virginie Borges-Cardoso, Natalia Pietrosemoli, Audrey Vallve, Stéphane Barron, Ophélie Jourjon, Orianne Lacroix, Aurélie Duthey, Manon Dirheimer, Maïlys Daniau, Catherine Legras-Lachuer, Gregory Jouvion, Caroline Carbonnelle, Hervé Raoul, Sylvain Baize

**Affiliations:** a Unité de Biologie des Infections Virales Emergentes, Institut Pasteur, Lyon, France; b Centre International de Recherche en Infectiologie (CIRI), Université de Lyon, INSERM U1111, Ecole Normale Supérieure de Lyon, Université Lyon 1, CNRS UMR5308, Lyon France; c Bioinformatics and Biostatistics Hub, Institut Pasteur, Université de Paris, Paris, France; d Laboratoire P4 INSERM–Jean Mérieux, INSERM US003, 69007 Lyon, France; e INSERM, Délégation Régionale Auvergne Rhône-Alpes, Bron, France; f ViroScan3D SAS, Trévoux, France; g Ecole Nationale Vétérinaire d’Alfort, Unité d’Histologie et d’Anatomie Pathologique, Maisons-Alfort, France; h Dynamic Research Group, Ecole Nationale Vétérinaired’Alfort, USC ANSES, Université Paris Est Créteil, Maisons-Alfort, France

**Keywords:** Lassa virus, cynomolgus monkeys, pathogenesis, immune responses, viral hemorrhagic fevers

## Abstract

The area of Lassa virus (LASV) circulation is expanding, with the emergence of highly pathogenic new LASV lineages. Benin recently became an endemic country for LASV and has seen the emergence of a new LASV lineage (VII). The first two outbreaks in 2014 and 2016 showed a relatively high mortality rate compared to other outbreaks. We infected cynomolgus monkeys with two strains belonging to lineage II and lineage VII that were isolated from deceased patients during the 2016 outbreak in Benin. The lineage VII strain (L7) caused uniform mortality. Death was associated with uncontrolled viral replication, unbalanced inflammatory responses characterized by increased concentrations of pro- and anti-inflammatory mediators, and the absence of efficient immune responses, resembling the pathogenesis associated with the prototypic Josiah strain in monkeys. The lineage II strain (L2) showed apparently lower virulence than its counterpart, with a prolonged time to death and a lower mortality rate. Prolonged survival was associated with better control of viral replication, a moderate inflammatory response, and efficient *T*-cell responses. Transcriptomic analyses also highlighted important differences in the immune responses associated with the outcome. Both strains caused strong inflammation in several organs. Notably, meningitis and encephalitis were observed in the cerebral cortex and cerebellum in all monkeys, independently of the outcome. Due to their apparently high pathogenicity, emerging strains from lineage VII should be considered in preclinical vaccine testing. Lineage II would also be beneficial in pathogenesis studies to study the entire spectrum of Lassa fever severity.

## Introduction

LASV is a mammalian Old-World arenavirus that causes Lassa fever, a hemorrhagic fever that leads to thousands of deaths each year and for which there is still no approved vaccine or treatment. All of West Africa is at risk of Lassa fever. Originally endemic to Nigeria, Sierra Leone, Liberia, and Guinea [1], LASV has recently emerged in additional countries, such as Mali, Côte d’Ivoire, Togo, and Benin [2–6]. Contamination generally occurs after contact with infected material from rodent reservoirs [7,8], which are believed to account for most human infections [9]. After an incubation period of one to three weeks, infected individuals first develop nonspecific signs and symptoms, such as fever and headache [10,11]. Other symptoms may appear during the first week, such as pharyngitis, cough, myalgia, abdominal pain, conjunctivitis, nausea, diarrhea, and vomiting. Survivors experience these symptoms during one to two further weeks before recovering but may develop long-term sequelae, including hearing deficits [12]. In severe cases, patients develop new symptoms after approximately one week, including edema, pleural and pericardial effusion, conjunctival and gingival bleeding, respiratory distress, and encephalopathy. Up to 50% of patients with severe cases then die from multi-organ failure and shock [10].

The pathogenesis of Lassa fever in humans can be fully recapitulated in cynomolgus monkeys and this model has been instrumental in our understanding of the pathophysiogenesis of this disease. The prototypic Josiah strain causes uniform lethality in cynomolgus monkeys [13,14]. After a short incubation period of three to six days, animals develop clinical signs, such as stress, fatigue, fever, and dehydration. Over the following days, animals usually develop other clinical signs, including bleeding, edema, and respiratory distress, and die within two weeks after infection. A similar disease is observed in monkeys infected with the strain Z-132, which belongs to the same lineage (lineage IV) as LASV Josiah [15]. Non-fatal infections can also be reproduced in cynomolgus monkeys infected with the lineage V strains Soromba-R and AV [13,15]. More recently, Stein et al. studied the pathophysiogenesis in cynomolgus monkeys of two strains from lineage III isolated from patients during the 2018 Nigerian outbreak that show different pathogenicity [16].

LASV presents an incredibly high diversity, with at least 7 different lineages based on their genetic differences. Many known or uncharacterized LASV strains co-circulate in the rodent reservoir, leading to the spillover of several strains during a single outbreak, possibly presenting differences in pathogenicity and antigenicity [9]. Their study in animal models is therefore essential to understand the pathophysiogenesis associated with each strain and to developing better countermeasures. This is particularly important for the development of LASV vaccines, which should offer cross-protection against the breadth of LASV strains circulating in West Africa [9,17]. We recently showed the efficacy of a Measles-based LASV vaccine against two strains from lineage II and VII that were isolated from deceased patients during the Benin 2016 outbreak [6,18]. Here, we describe the pathophysiogenesis associated with these two strains in unvaccinated cynomolgus monkeys in more detail.

## Results

### LASV virus isolation

**Figure 1.**
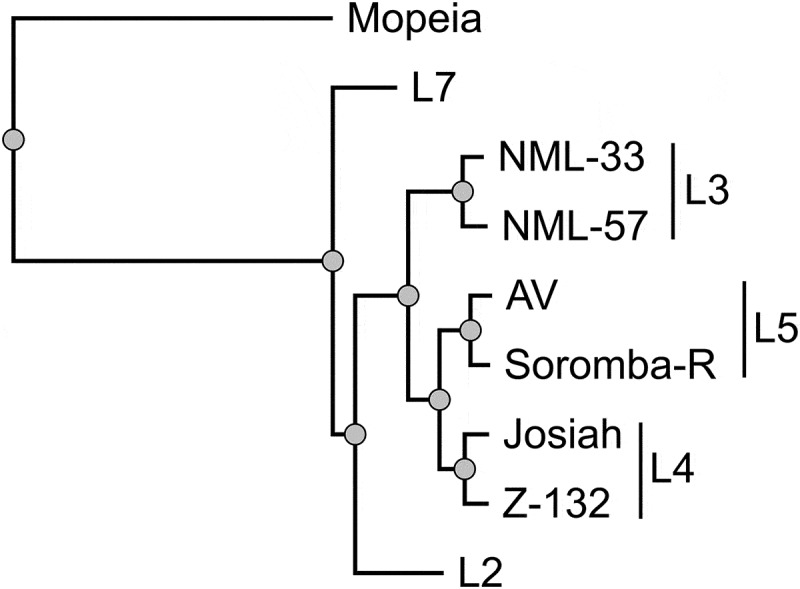
Phylogenetic analysis of the LASV strains tested in cynomolgus monkeys. the tree was inferred using the PhyML Smart Model Selection [21] general time-reversible plus gamma plus proportion of invariable sites model, performed on the L gene of the different LASV strains. The L gene of the Mopeia virus strain an 20410 was used to root the tree. as the sequence of the Z-132 strain is not publicly available, we used the sequence of Z-148, which is genetically similar [15]. The LASV lineage is indicated on the right.

The lineage II strain BEN-16081 was isolated on Vero E6 cells from a blood specimen of a 32-year-old female merchant traveling from Lagos who died in a Porto Novo hospital in January 2016 [6]. The lineage VII strain BEN-16131 was isolated on Vero E6 cells from a blood sample of a 25 year-old female merchant working in Tchaourou who died in April 2016. Strain BEN-16081 is called L2 and strain BEN-16131 is called L7 in this study. After isolation on Vero E6 cells, a pre-stock was produced on Vero E6 cells by infecting cells at a multiplicity of infection of .01. Viruses were collected three days later and cleared supernatants were titrated by focus forming assays, reaching titers of 3 × 10^7^ FFU/mL for L2 and 8.5x10^6^ FFU/mL for L7. We performed a phylogenetic analysis on the L gene of several LASV strains tested in cynomolgus monkeys, the gold standard for the study of LASV pathogenesis in animal models. This analysis included two strains from lineage IV (Josiah [14] and Z-132 [15]) two strains from lineage V (AV [13,19] and Soromba-R [15]), and two strains from lineage III (NML-33 and NML-57 [16]), in addition to the aforementioned strains L2 and L7. Strain 0043/LV/14 from lineage II was not included in the analysis, as the sequence is not available [20]. As expected, strains belonging to the same lineages clustered together on a phylogenetic tree (Figure 1). Among all strains, L2 and L7 were the most phylogenetically distant from the prototypic strain, Josiah.

### Pathogenesis of the L2 and L7 strains in cynomolgus monkeys

Two groups of three monkeys were infected by a subcutaneous injection of 3,000 FFU of LASV strain L7 or L2 diluted in 500 µL of PBS. Clinical manifestations were then monitored on a daily basis and sampling was performed every three days for the first two weeks and then once a week for the following two weeks. As previously reported [18], all animals experienced weight loss and decreased tonus between day 3 and day 9 (Table 1), accompanied by fever for two animals in each group. Between days 10 and 15, L7-infected animals developed more pronounced clinical signs, including apathy, shivering, and hypothermia, and all had to be euthanized between day 12 and day 15. Animal L2.1 showed epistaxis and diarrhea after day 10 and was euthanized on day 12. The two other animals only showed low tonus and dehydration. Animal L2.3 survived until day 20, showing balance issues and apathy on the day of euthanasia. Animal L2.2 survived until the end of the protocol and did not show any clinical signs after day 15.

**Table 1.** Clinical, virological, and immunological findings in LASV-challenged monkeys. for the plasma concentrations of alanine transferase (ALT), aspartate transferase (AST), and C-reactive protein (CRP), + indicates 2 < fold increase (FI) < 5, ++ 5 <fi < 10, and +++ FI > 10. the day of observation is indicated within the parentheses. for viremia, + indicates a plasma titer between 10^2^ and 10^4^ FFU/mL, ++ between 10^4^ and 10^5^ FFU/mL, and +++ > 10^5^ FFU/mL. LASV strain-specific IgG titers were measured by ELISA and + corresponds to titers below 1/1000; ++ between 1/1000 and 1/4000, and +++ > 1/4000. LASV strain-specific T-cell responses were measured by intracellular cytokine staining and the percentage of cytokine-positive (expressing IFNγ and/or TNFα) CD4+ and CD8+ T cells is indicated by + when between .1 and .2, ++ between .2 and .3, and +++ > .3. the time of death corresponds to the day of euthanasia (clinical score above 15 or terminal endpoint), surviving animals were euthanized at the end of the protocol on day 28

	Day 1-2	Day 3-9	Day 10-15	Day 16-21	Death
L7.1	Stress	Weight loss, low tonus, fever,	Weight loss, hypothermia,		Day 12
		ALT++ (9), AST++ (9), CRP+ (3),	ALT+++ (12), AST+++ (12), CRP++ (12),		
		Lymphopenia,	Lymphopenia,		
		Viremia +, T CD4++++ (6)	Viremia +++, IgG ++		
L7.2	Stress	Weight loss, low tonus, fever,	Apathy, shivering, prostration, pain, hypothermia (15), ALT +++ (12),		Day 15
		Lymphopenia,	AST +++ (12), CRP +++ (15), lymphopenia, viremia +++, IgG ++,		
		Viremia +,	T CD8+ +		
		T CD8+ + (6), CD4+ + (6)			
L7.3	Sress	Weight loss, low tonus,	Apathy, shivering, hypothermia,		Day 14
		ALT +, AST ++ (9), CRP +++ (3),	ALT +++ (12), AST +++ (12), CRP +++,		
		Lymphopenia, viremia ++,	Lymphopenia, neutropenia,		
		IgG + (9), T CD8+ + (6), CD4+ + (6)	Viremia +++, IgG +++		
L2.1	Stress	Weight loss, low tonus, fever,	Nosebleed, diarrhea,		Day 12
		AST + (9), CRP +++ (3),	AST ++ (12), CRP +++, lymphopenia,		
		Lymphopenia, viremia +	Viremia +++, IgG +++		
L2.2	stress	Weight loss, low tonus,	Low tonus, dehydration,	IgG +++,	Survived
		AST ++ (9), CRP + (9)	AST + (12), CRP +++ (12), lymphopenia,	T CD8+ ++	
		Lymphopenia	Viremia ++ (12), IgG +++ (15),		
			T CD8+ ++ (15), CD4+ ++ (15)		
L2.3	Stress	Weight loss, low tonus, fever,	Low tonus, dehydration,	Unsteadiness, apathy, viremia +++, IgG +++ (20)	Day 20
		CRP + (6), lymphopenia,	ALT ++ (15), AST ++ (15), CRP ++ (12),		

As previously described [18], all animals showed increased plasma concentrations of alanine aminotransferase (ALT) and aspartate aminotransferase (AST) between day 9 and day 15 or the day of death, as well as increased plasma concentrations of C-reactive protein (CRP) between day 3 and day 15 or the day of death. All animals experienced marked lymphopenia between day 3 and day 12, affecting CD4+ and CD8+ T cells, as well as B cells and NK cells (Table 1 and Figure S1). After day 12, the number of circulating T and B cells, NK cells, monocytes, and granulocytes increased in the surviving L2.2 animal (Figure S1).

As previously reported [18], viremia was detected in all L7-infected animals as early as day 6 post-challenge and increased until the time of death (Table 1). Viremia was detected on day 9 in the L2-infected animals and increased until the time of death in animal L2.1, which died on day 12 (Table 1). Viremia increased more slowly in animal L2.3, which survived until day 20, but reached titers above 10^5^ FFU/mL. Viremia was only detected between day 9 and day 12 in animal L2.2, which survived the L2 infection (Table 1). At the time of necropsy, we collected several organs and measured the infectious titers in each (Sup. Table S1). We detected infectious virus in the lymph nodes, spleen, liver, lung, kidney, adrenal gland, bladder, brain, and cerebellum of all L7-infected animals, except in the mesenteric lymph nodes of L7.1 and in the cerebellum of L7.3. Organs from animal L2.1 also contained infectious virus in amounts comparable to those observed in the L7 animals. Infectious titers measured in the organs from L2.3 were relatively low relative to those observed for L2.1 and no virus was found in the bladder. Animal L2.2 only showed a very low infectious titer in the spleen, just above the limit of detection.

We also assessed the humoral and cellular responses in all animals (Table 1). As reported previously in more details [18], LASV-specific IgG were detected in all L7 animals at day 9 and in all L2 animals at day 12 and reached high titers at the time of death. TNFα-producing LASV-specific CD4+ T cells were detected in L7 animals at day 6 post challenge but were not detected later. L2 animals did not develop LASV-specific T cell responses except for the surviving animal who produced IFNγ-producing LASV-specific T CD4+ and T CD8+ cells by day 15 post challenge, and the T CD8+ response was still detected at day 28.

### Cytokine and chemokine responses to LASV infection

**Figure 2.**
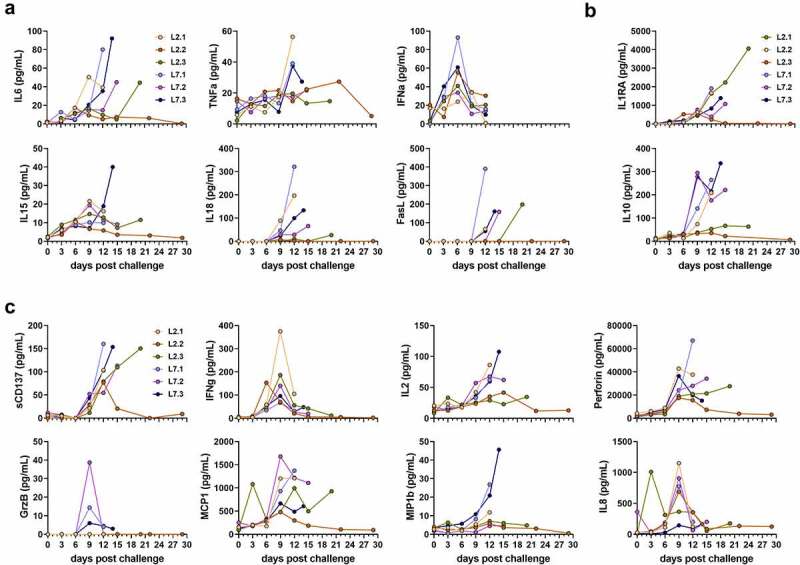
Quantification of soluble mediators in the plasma according to the time after LASV infection. Plasma concentrations of (a) pro-inflammatory cytokines, (b) anti-inflammatory cytokines, (c) T-cell response-related mediators, and (d) chemokines at various times post-challenge. IFN-α plasma concentrations were measured by ELISA. All other plasma concentrations were measured by Luminex assays.

We measured the plasma concentrations of several cytokines and chemokines in the days following challenge. Plasma concentrations of pro-inflammatory IL-6, IL-18, and FasL increased for all infected monkeys in the days preceding death, except for the surviving animal L2.2 (Figure 2). Concentrations of TNFα were also elevated at the time of death in the animals that died before day 14 but remained stable in the others. We observed a peak in the IFNα concentration for almost all animals on day 6 post-challenge. Elevated concentrations of IL-15 were also detected in the plasma of animal L7.3 at the time of death. The plasma concentrations of the anti-inflammatory cytokines IL-1RA and IL-10 increased steadily between day 9 and the time of death for all animals, except L2.2 (Figure 2). The increase in IL-10 plasma concentrations was also modest for L2.3, which survived until day 20, relative to that of the other animals, which died earlier. The plasma concentrations of several soluble mediators involved in cytotoxic activity and regulation of the immune response were also affected after challenge. The concentration of sCD137 increased for all animals between day 9 and the day of death, except for L2.2, which showed a peak on day 12, which then returned to the basal value (Figure 2). We also detected a peak in the release of IFNγ on day 9 for all animals, except L2.2, for which the IFNγ peaked earlier, on day 6 (Figure 2). The IL-2 concentrations rose until the time of death for all animals that died before day 15 but remained low for animals L2.2 and L2.3 (Figure 2). In animal L7.1, the concentration of perforin was the highest at the time of death, whereas perforin concentrations increased considerably by day 9 and then tended to stabilize or even decrease for the other animals (Figure 2). A modest and transient increase in the plasma concentration of granzyme B was only noted in the L7-infected animals on day 9 (Figure 2). The chemokines MCP-1 and IL-8 were released on day 3 at high concentrations in the plasma of L2.3, whereas these chemokines were released on day 9 and only transiently for IL-8 in the other animals (Figure 2).

### Transcriptomic profiles after LASV infection

**Figure 3.**
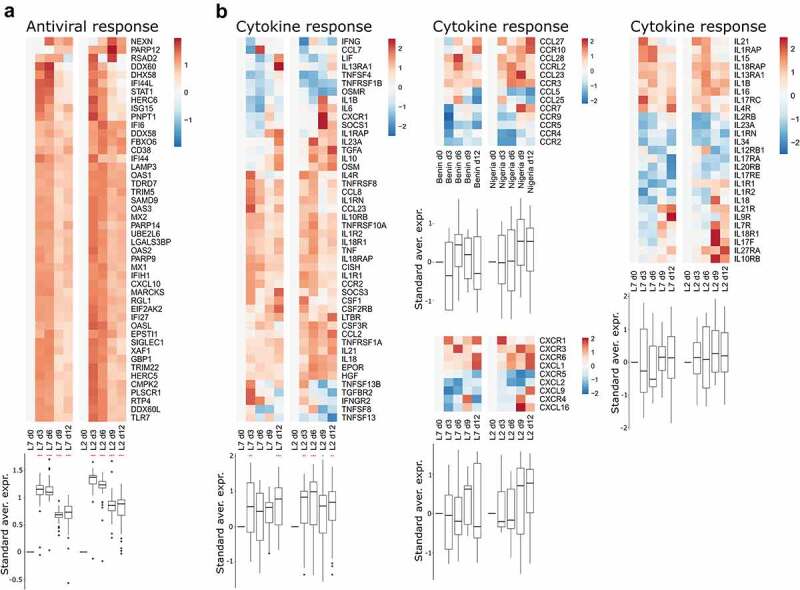
Transcriptomic analyses of PBMCs from infected animals. Gene expression heatmap of two gene sets: (a) antiviral response and (b) cytokine response. the standardized average expression (Standard aver. expr.) of the genes for each gene set was plotted against the time after challenge and normalized to day 0. for each gene set, a two-way ANOVA, adjusted for the viral strain and the day, was fitted to the standardized (centered and scaled) gene expression to summarize the global direction of regulation of the gene sets. Comparisons of average gene expression between day 0 and other days were performed using contrasts of the linear model (post-hoc Tukey HSD test). Red asterisks indicate significant differences from the expression on day 0 *P ≤.05, **P ≤.01, ***P ≤.001.

**Figure 4.**
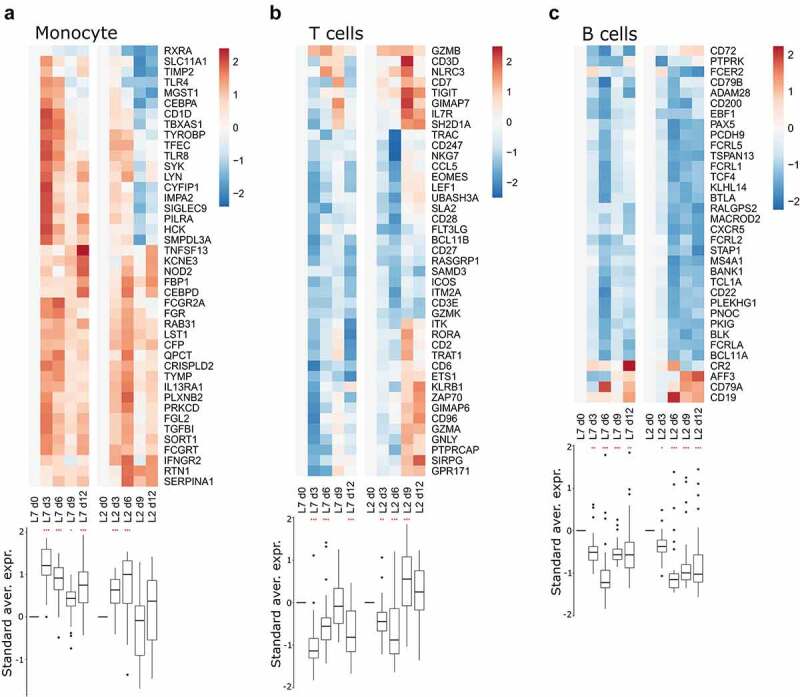
Transcriptomic analyses of PBMCs from infected animals. Gene expression heatmap of three gene sets: (a) monocyte response, (b) T-cell response, and (c) B-cell response. the standardized average expression (Standard aver. expr.) of the genes for each gene set was plotted against the time after challenge and normalized to day 0. for each gene set, a two-way ANOVA, adjusted for the viral strain and day, was fitted to the standardized (centered and scaled) gene expression to summarize the global direction of regulation of the gene sets. Comparisons of average gene expression between day 0 and other days were performed using contrasts of the linear model (post-hoc Tukey HSD test). Red asterisks indicate significant differences from the expression on day 0 *P ≤.05, **P ≤.01, ***P ≤.001.

We performed RNA-seq on RNA extracted from peripheral blood mononuclear cells (PBMCs) collected every three days for 12 days after challenge with each viral strain. We then analyzed the transcriptomic response using predefined gene sets corresponding to various responses. The differential expression of selected genes was evaluated relative to the expression on day 0, at the time of challenge (Figures 3 and 4). We detected strong upregulation of genes related to the antiviral response on days 3 and 6 in the PBMCs of animals infected with either strains L7 or L2 (Figure 3). Their expression tended to diminish by days 9 and 12, but was still significantly elevated relative to day 0. Many genes related to the cytokine response were also upregulated as soon as day 3 in both groups and the expression of many cytokines remained elevated until day 12 (Figure 3). The induction of certain cytokine genes was more pronounced in the L2- than L7-infected animals, notably *CCL2*, *IL21*, and *IL18*, for which upregulation was observed as soon as day 6 post-challenge (Figure 3, left panel). On day 9, expression of the genes *IL7 R, IL18R1, IL17F, IL27RA*, and *IL10RB* was also higher relative to day 0 for the L2-infected animals than on day 9 for the L7-infected animals (Figure 3, right panel). In the L7 group, genes related to the monocyte response were strongly upregulated on day 3 and their expression then diminished over time but remained significantly higher than on day 0 (Figure 4). In the L2 group, the monocyte gene set was less strongly upregulated, the upregulation peaked later, on day 6, and then many genes of this gene set were downregulated (Figure 4). Consequently, this gene set was no longer upregulated on day 9 and day 12 relative to day 0. Genes involved in the T cell-response were rapidly downregulated in both groups (Figure 4). In the L7-infected animals, downregulation was less marked on days 6 and 9 but still significantly different from their expression on day 12. In the L2-infected animals, we observed strong upregulation of the gene set on day 9 and the expression of many genes remained elevated up to day 12. Significant downregulation of the overall expression of genes involved in the B cell response was observed for both groups at every time point post-challenge and was particularly marked on day 6 (Figure 4).

### Histopathology of LASV L2 and L7 infection

A histopathological analysis was carried out in several lymphoid and systemic organs that are known to play a key role in Lassa pathogenesis. L7-infected monkeys showed similar pathology in most organs except the liver where slight differences were observed between different animals; we, therefore, present the data for only one animal in the figures (except for the liver). Similarly, the histological findings of fatally L2-infected animals were consistent; hence we show the data for one deceased animal in comparison to L2.2, which was the only non-fatal case of Lassa fever in this study. The control animals were historical controls from our previous studies [13].

**Figure 5.**
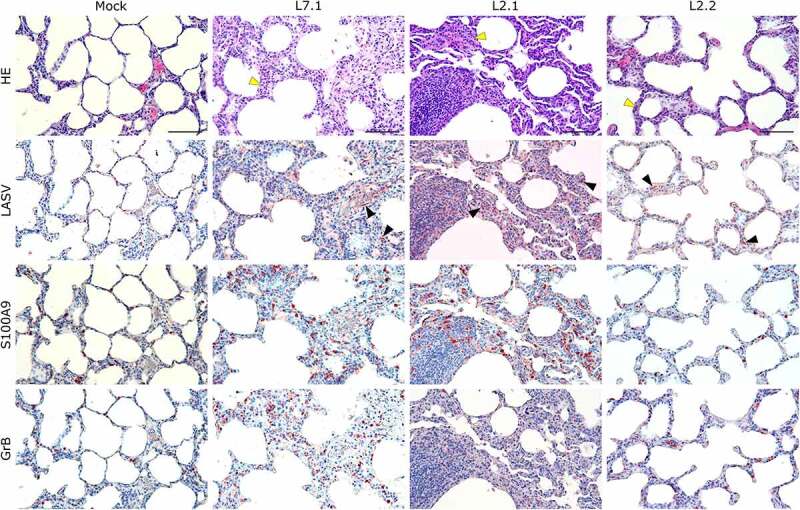
Comparative histopathology of the lung after challenge with L7 or L2 LASV strains. HE: hematoxylin-eosin coloration. Yellow arrowheads show severe (L7.1 and L2.1) or mild (L2.2) septal thickening. LASV: immunostaining of LASV glycoprotein-2c. Black arrowheads show infected cells. S100A9: immunostaining of neutrophils. GrB: immunostaining of cytotoxic cells. Scale bar: 100 µm.

**Figure 6.**
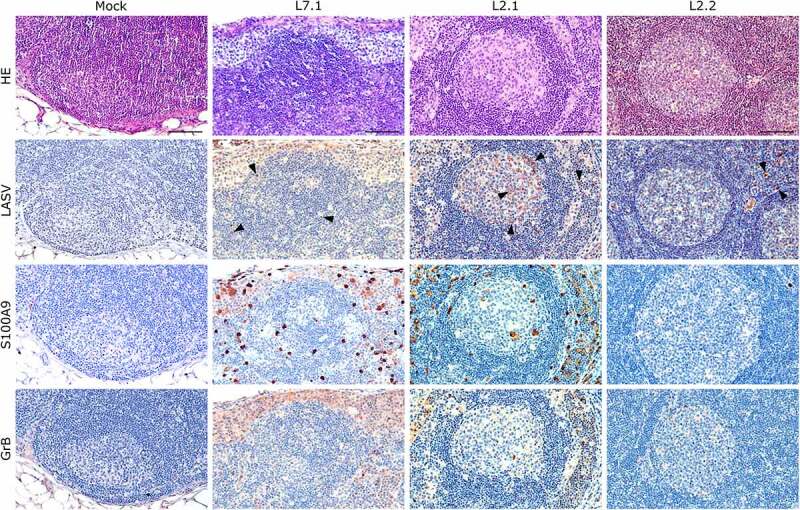
Comparative histopathology of a mesenteric lymph node after challenge with L7 or L2 LASV strains. HE: hematoxylin-eosin coloration. LASV: immunostaining of LASV glycoprotein-2c. Black arrowheads show infected cells in the mantle, the germinal center and the cortical sinus. S100A9: immunostaining of neutrophils. GrB: immunostaining of cytotoxic cells. Scale bar: 100 µm.

**Figure 7.**
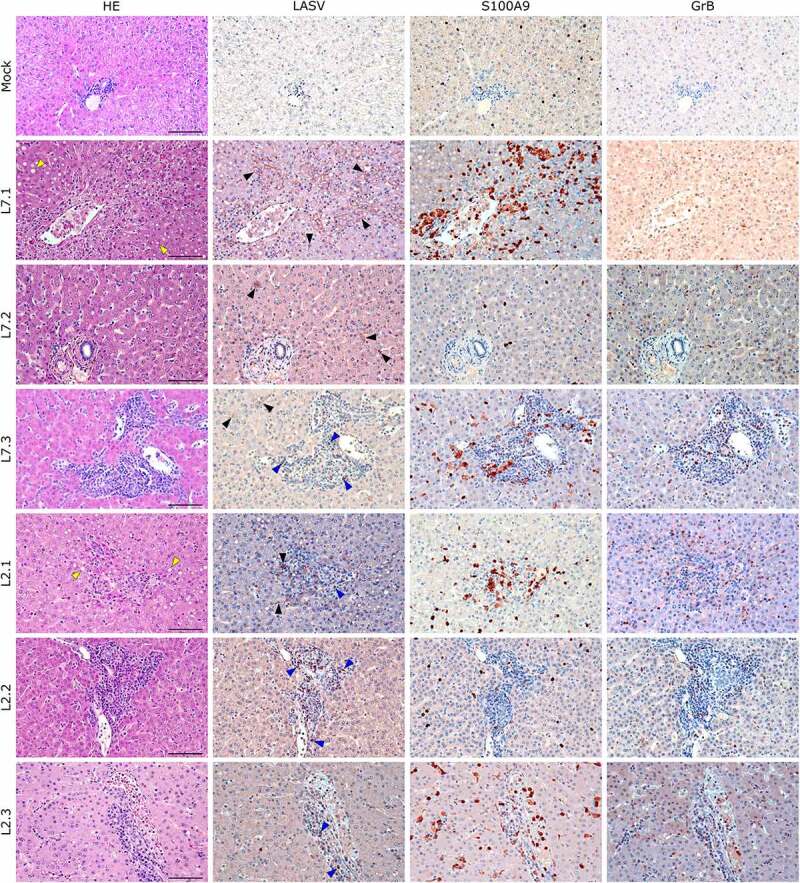
Comparative histopathology of the liver after challenge with L7 or L2 LASV strains. HE: hematoxylin-eosin coloration. Yellow arrowheads show signs of hepatic steatosis. LASV: immunostaining of LASV glycoprotein-2c. Black arrowheads show infected hepatocytes and interstitial cells, blue arrowheads show infected immune infiltrating cells. S100A9: immunostaining of neutrophils. GrB: immunostaining of cytotoxic cells. Scale bar: 100 µm.

Notably, fatally infected animals exhibited histologic lesions that reflected the respiratory distress noted clinically, with considerable thickening of the alveolar septum and advanced interstitial pneumonia, regardless of the strain they were infected with (Figure 5). Interestingly, animal L2.2 showed only minor septal thickening, although the alveolar space was still reduced relative to mock-infected animals. Furthermore, we observed signs of disruption of the lymphoid tissues of L7-infected animals (Figures 6 and S2). In the mesenteric lymph nodes, secondary lymphoid follicles were essentially unstructured, with little delimitation between the mantle and cortical sinus and almost no germinal center (Figure 6). The splenic marginal zone of these animals was also widely disrupted and sometimes entirely destroyed (Figure S2). Infections with L2 and L7 strains induced the appearance of tingible body macrophages in the lymphoid follicles of the lymph nodes and spleen (Figures 6and S2). Hepatitis, nephritis, encephalitis, and meningitis were detected in all animals, including L2.2, as mononuclear cell infiltration was found in all organs, demonstrating a general inflammatory state caused by both strains of LASV. Finally, hepatic steatosis was identified in animals L7.1 and L2.1 (Figure 7).

**Figure 8.**
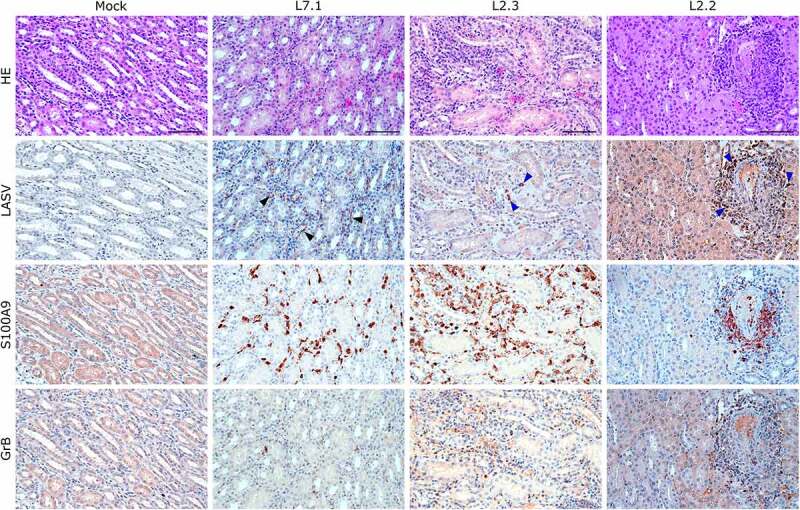
Comparative histopathology of the kidney after challenge with L7 or L2 LASV strains. HE: hematoxylin-eosin coloration. LASV: immunostaining of LASV glycoprotein-2c. Black arrowheads show infected endothelial cells, blue arrowheads show infected immune infiltrating cells. S100A9: immunostaining of neutrophils. GrB: immunostaining of cytotoxic cells. Scale bar: 100 µm.

We identified the infected cells in all organs we investigated for both L2 and L7. Hepatocytes showed a very wide range of infection patterns (Figure 7). Indeed, we detected no infected hepatocytes in L2.2 and L2.3 and only very small and sparse clusters of infected hepatocytes in L2.1, L7.2, and L7.3, whereas infection was widespread throughout the hepatic tissue in L7.1. Nevertheless, infected cells were identified within the periportal infiltrates of mononuclear cells in L2-infected animals. Endothelial cells were very permissive to L7 infection, as identified in the kidneys (Figure 8) and lungs (Figure 5). As in the liver, we detected infected immune cells in the perivascular infiltrates of the kidney during L2 infection (Figure 7). In the spleen, L7-infected cells were found both in the lymphoid follicles (marginal zone, mantle, and germinal center) and the red pulp, whereas L2 infection appeared to remain limited to the marginal zone and germinal centers, and was only sparse in the red pulp (Figure S2). In the mesenteric lymph node, fatal L2 infection was characterized by a significant amount of viral material in the germinal centers, whereas the few infected cells were disseminated in the sinuses and in the medulla in other conditions (Figure 6).

**Figure 9.**
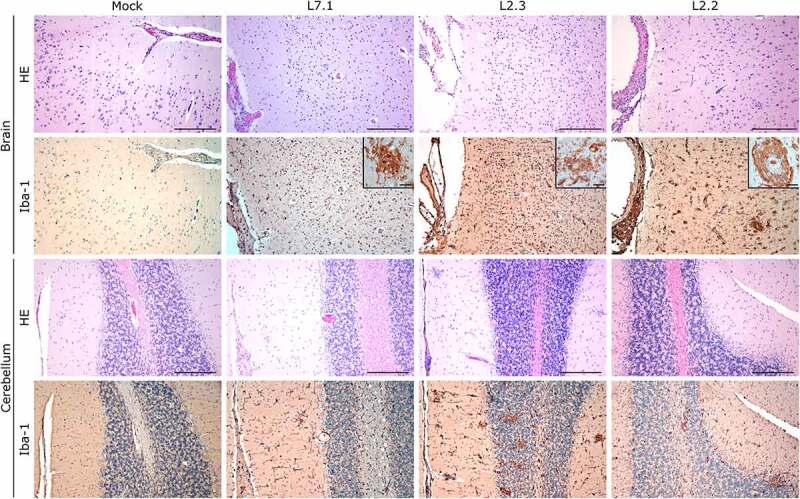
Comparative histopathology of the brain and cerebellum after challenge with L7 or L2 LASV strains. HE: hematoxylin-eosin coloration. Iba-1: immunostaining of the microglia (dim in normal microglia, bright inactivated microglia). Scale bar: 250 µm. Scale bar in magnifications: 25 µm.

S100A9 immunostaining also showed widespread neutrophilic infiltration in the lungs, liver, kidneys, and adrenal glands during fatal infection (Figures 6–8 and S3), which was not limited to the clusters of infiltrated immune cells but widespread throughout the functional areas of each organ. Conversely, the functional areas of systemic tissues of L2.2 remained mostly free of neutrophil invasion, although some neutrophils were found in perivascular infiltrates only. Many neutrophils were also detected in the sub-capsular and cortical sinuses of the lymph nodes of fatally infected animals, whereas L2.2 showed no sign of neutrophil infiltration in the lymph nodes, similar to the mock-infected animals (Figure 6). Deceased animals also showed widespread neutrophil invasion of the splenic marginal zone and red pulp (Figure S2). We detected a considerable number of granzyme B-positive cells in the alveolar septum of L7-infected animals (Figure 5). All infected animals showed a moderate increase in the number of cytotoxic cells in all other organs except the adrenal glands, regardless of the tissue or strain. Importantly, microglial activation – detected by enhanced Iba-1 immunostaining and reduced size of the dendrites – in all layers of the brain and cerebellum was strong and similar in all infected animals (Figure 9). We identified microglial nodules both in the white and gray matter of the brain (Figure 9, Brain, see magnifications) and in the molecular and granular layers, as well as in the white matter of the cerebellum (Figure 9, Cerebellum).

## Discussion

Here we describe the pathophysiogenesis of two LASV strains, L2 and L7, in the cynomolgus monkey model. These two strains were isolated from samples collected from patients who died from Lassa fever during the 2016 outbreak in Benin [6]. In monkeys, these two strains showed different pathogenicity. L7-infected animals all died within 15 days, whereas one L2-infected animal survived the infection and another died only on day 20. Strain L7 appears to be as pathogenic as strains from lineage IV, Josiah and Z-132, which usually cause uniform lethality within 15 days in cynomolgus monkeys [13–15,18,22,23]. On the contrary, the pathogenicity of strain L2 is closer to that of strains Soromba-R and AV from lineage V in monkeys [4,24]. Indeed, the challenge of three macaques with 10^4^ FFU of LASV Soromba-R led to one death on day 12, one death on day 18, and a third animal survived the challenge [15]. Similarly, the challenge of three monkeys with 10^3^ FFU of LASV AV led to heterogeneous mortality, with one animal dying on day 16, a second on day 21, and a third surviving the infection [19]. In a recent study, four infected monkeys all survived infection with a similar dose of LASV AV [13]. These observations suggest that strains from lineage V, which have, thus far, caused only two known human deaths [24,25], are less pathogenic than strains from other lineages. But it is not the case in all lineages. While L2 appeared less pathogenic than L7 in monkeys, LASV strain 0043/LV/14 from Lineage II is as pathogenic as strains from lineage IV and VII in monkeys, causing the death of infected animals within 15 days after a challenge with 10^3^ FFU [20]. Very recently, cynomolgus monkeys were infected with 10^4^ TCID_50_ of two strains belonging to lineage III, isolated from patients during a 2018 Nigerian outbreak [16]. Strain NML-33 caused the death of the four infected monkeys within 14 days. Strain NML-57 only caused one death on day 16 and the other three animals survived the infection. These observations underscore the necessity to study a wide variety of LASV strains belonging to different lineages to understand the pathophysiogenesis of Lassa fever and the various outcomes associated with an infection. This is particularly important, as recent analyses have demonstrated that many LASV strains belonging to different lineages can co-circulate during a single outbreak [9].

Independently of the outcome, all animals infected with L7 and L2 developed signs of disease and presented hallmarks of LASV infection between the day of challenge and day 9. These included stress, loss of weight and tonus, fever, an increase in AST and ALT levels, and lymphopenia. Viremia appeared earlier and increased more rapidly in L7-infected animals, suggesting that L7 and L2 may show different virulence. At the time of necropsy, the titers found in organs were higher overall in L7- than L2-infected animals. Histopathological analyses showed viral replication to be mostly restricted to endothelial cells, except for the liver, where hepatocytes were also infected. The health of L7-infected animals considerably declined after 10 days and they showed severe hypothermia at the time of death, as observed in Josiah-challenged animals [13,23]. In the L2 group, animal L2.1, which died on day 12, showed epistaxis, which may indicate a coagulation disorder, as observed in NML-33-infected animals [16]. Animal L2.3 was euthanized on day 20 after showing serious balance issues, which may suggest neurological illness. Hearing deficits, a common complication of Lassa fever, have been described in Josiah-infected monkeys [26] and may explain, at least in part, the balance issues. We were not able to evaluate potential hearing deficits of infected monkeys in our BSL-4 facilities. At the time of necropsy, we observed a strong neuro-inflammation in all monkeys regardless of the strain or the outcome, characterized by meningitis, encephalitis, diffuse microglial activation and formation of microglial nodules in the brain and the cerebellum. Interestingly, the animal L2.2 that survived and had no detectable viremia after day 12 still exhibited histological signs of encephalopathy at the terminal point of the study, suggesting that neurological disorder can last for a prolonged time after recovery from LASV infection. Multifocal gliosis and inflammation of the brain and the cerebellum have also been described during the course of non-fatal Soromba-R – lineage V – infection [15]. In humans, a third of the patients enrolled in a large Nigerian cohort presented central nervous system manifestations during LASV illness and treatment, and encephalopathy was strongly associated with fatal issue [27]. These previous findings, combined with the extensive neuro-inflammation we describe in this study suggest that the neurological manifestations caused by Lassa fever should not be underestimated.

Deceased macaques infected by L2 or L7 all exhibited acute respiratory distress. Although we lack specific clinical analyses – volumetric monitoring, lung radiography – to characterize the respiratory syndrome, histological observations show evidence of advanced interstitial pneumonia, with diffuse infiltration of mononuclear cells and thickening of the alveolar septa. Similar pulmonary manifestations were described by previous studies investigating the pathophysiology of various strains of LASV, notably from lineage III (NML-33), IV (Josiah) and V (Soromba-R) [13,15,16]. In Soromba-*R*-infected animals, Safronetz and colleagues partly attributed these clinical signs to the strong increase of TNF-α observed during the course of the disease and which is known to correlate with vascular permeability [15,28]. More recently, animals infected with NML-33 and NML-37 strains also develop a strong pulmonary disease in absence of TNF-α secretion [16]. In our study, the five animals who died before the end of the experiment presented pulmonary manifestations but only three of them presented a terminal increase in plasmatic levels of TNF-α. Therefore, TNF-α secretion was probably not the major cause of the acute lung pathology we described, but this pathology was strongly associated with fatal outcome. This corroborates results obtained with a large human cohort dataset showing a significant association between respiratory distress and outcome during Lassa fever [27]. Altogether, these recent findings tend to show that the impairment of the respiratory function might be an unneglectable effect of LASV infection, although more investigations are to be led to better characterize this respiratory syndrome.

The surviving animal, L2.2, likely survived after mounting an efficient cellular response. Indeed, L2.2 was the only animal to develop LASV-specific CD8+ and CD4+ T-cell responses between day 10 and day 15. Transcriptomic analyses also showed that genes associated with the T-cell response were only upregulated in L2-infected animals starting from day 9 post-infection. These observations are consistent with the crucial role of the T-cell response in the protection of human survivors or immunized monkeys [19,23,29–32]. The antibody response did not presumably participate in protection, as all L2- and L7-infected animals produced similar levels of LASV-specific IgG before death. At the terminal stage of the disease, we observed strong dysregulation of the inflammatory response in all animals that died before day 15, with a substantial release of pro-inflammatory and anti-inflammatory mediators that was particularly marked in the L7-infected animals. On the contrary, animals L2.2 and L2.3 showed a more balanced inflammatory response, similar to that observed in AV-infected animals [13]. The cells responsible for the release of soluble mediators are likely activated monocytes and macrophages. PBMCs showed a strong activated monocyte signature in the L7-infected animals as early as day 3 post-infection and until day 12, and the overall activation of monocyte genes inversely mirrored the expression of T cell-related genes. In L2-infected animals, the monocyte response was only upregulated between days 3 and 6, and after day 6, we detected the upregulation of T cell-related genes, which persisted until day 12. Thus, the strong activation of monocytes and macrophages producing high amounts of cytokines may explain, at least partially, the absence of proper T-cell responses during fatal Lassa fever, as proposed previously for Josiah-infected monkeys [13]. The combined use of immunomodulatory and antiviral molecules may therefore be a strategy to treat Lassa fever patients. Recently, the JAK/STAT inhibitor ruxolitinib was shown to prevent terminal hypothermia and pleural effusion in a mouse model of arenavirus hemorrhagic fever [33], without altering viral replication, presumably by dampening the cytokine response [34].

In the absence of efficient treatment, vaccination remains the most viable means to control LASV infection during outbreaks. Many vaccines are being developed and at least two are already in clinical trials [20,22,26,35,36]. According to the World Health Organization, a LASV vaccine should protect against LASV lineages I-IV. Our results demonstrate that strains from lineage VII should also be considered in vaccine testing, as they may be at least as pathogenic as the historical strains from lineage IV used to generate all vaccines and are highly divergent from them. Our results also support the testing of new emerging LASV strains in animal models to understand specific aspects of their pathogenesis and to develop better countermeasures.

## Materials and methods

### Study design

Animals were subcutaneously (SC) challenged with 3,000 focus-forming units (FFU) of LASV BEN-16081 lineage II (L2) [6] or 3,000 FFU of BEN-16131 lineage VII (L7) [6]. After challenge, animals were monitored and were attributed clinical scores on the basis of observed clinical signs, body temperature, body weight, feeding, dehydration and behavior, as previously described [18,23]. A clinical score of 15 or above was the limit point for euthanasia in order to limit animal suffering. Animals were anesthetized every three days until day 15 then once a week until the end of the experiment at day 28. During anesthesia, blood, oral and nasal swabs, and urine were collected in parallel of the medical exam. All procedures were approved by the Comité Régional d’Ethique en Matière d’Expérimentation Animale de Strasbourg (2018100414445313) and the Comité Régional d’Ethique pour l’Expérimentation Animale Rhône Alpes (2018101211262022).

### Cell cultures, virus, and infections

Vero E6 cells were grown in Glutamax Dulbecco Modified Eagle’s Medium (DMEM, Life Technologies) supplemented with 5% fetal bovine serum (FBS) and 0.5% penicillin-streptomycin. Stocks of LASV BEN-16081 lineage II (L2) and BEN-16131 lineage VII (L7) were produced and titrated by focus-forming assay on Vero E6 cells. Briefly, Vero E6 cells were infected for 1 h with decimal dilutions of the viral stocks then overlaid with carboxy-methylcellulose diluted in DMEM. Seven days later, cells were washed, fixed, permeabilized and stained with mouse anti-LASV antibodies (kindly provided by the CDC, Atlanta) and goat anti-mouse antibodies conjugated to alkaline phosphatase. Infectious foci were revealed by incubation with the NBT/BCiP substrate (Thermofisher) and counted manually. Inoculums were prepared in phosphate buffered saline (PBS). Animals received 3,000 focus-forming units (FFU) by the SC route. Viral titers in plasma and organs were measured by focus-forming assay on Vero E6 cells.

### Quantitative RNA analysis

Quantitative PCR for viral RNA was performed with the SensiFAST Probe No-ROX One-Step kit (Bioline), and NP-specific primers and probes for the L2 and L7 strains. Quantification was done thanks to NP-specific synthetic RNA standards as previously described [37].

### T-cell activation assay

LASV-specific T-cells against L2 and L7 strains were determined as described elsewhere [18]. Briefly, fresh whole blood was incubated with overlapping GPC or NP peptides covering the full sequence of the proteins (15 amino acid long, 11 residues overlapping, 1 µg/ml each) and anti-human CD28 and anti-human CD49d antibodies (2 µg/ml, BD Biosciences) and Brefeldin A (10 µg/ml, Sigma-Aldrich) for 6 h at 37°C. Staphylococcus enterotoxin A (SEA) (1 µg/ml, Sigma-Aldrich) and PBS were used as positive and negative control of activation, respectively. PBS-ethylene diamine triacetic acid (EDTA) (2 mM final concentration) was then added to the samples before staining with APC mouse anti-human CD3 (clone SP34-2), Alexa Fluor^R^ 700 mouse anti-human CD4 (clone L200), and APC-H7 mouse anti-human CD8 (clone SK1) (BD Biosciences). Samples were then treated with PharmLyse (BD Biosciences) to lyse red blood cells. Cells were fixed and permeabilized using the FoxP3 staining buffer set (Miltenyi Biotec) before intracellular staining with antibodies to IFNγ (clone B27, PE) and TNFα (clone Mab11, Pe-Cy7) (BioLegend). Cells were analyzed by flow cytometry using a 10-color Gallios cytometer (Beckman Coulter). Data were analyzed using Kaluza 2.1 software (Beckman Coulter).

### Hematology and biochemistry

The number of circulating CD8 and CD4 T cells, B cells, NK cells, monocytes, and granulocytes was determined by flow cytometry using the following antibodies: V540 mouse anti-human CD56 (clone B159), V500 mouse anti-human CD3 (clone SP34-2), FITC Mouse anti-NHP CD45 (clone D058-1283), PE mouse anti-human CD10 (clone HI10α), PE-Cy^TM^7 mouse anti-human CD20 (clone 2H7), Alexa Fluor^R^ 700 mouse anti-human CD4 (clone L200), APC-H7 mouse anti-human CD8 (clone SK1) all from BD biosciences and APC mouse anti-human NKp80 (clone 4A4.D10) from Miltenyi Biotec. Cells were analyzed by flow cytometry using a 10-color Gallios cytometer (Beckman Coulter). Data were analyzed using Kaluza 2.1 software (Beckman Coulter). Plasma concentrations of ALT, AST, and CRP were measured using a Pentra C200 analyzer (Horiba Medicals).

### Enzyme-Linked immunosorbent assays

LASV specific IgG antibody titers were determined as described elsewhere [18]. Briefly, polysorp plates (Nunc) were coated overnight with a lysate of LASV-infected (L2 or L7) or mock-infected Vero E6 cells as positive and negative antigens, respectively. Plates were then blocked with PBS/2.5% bovine serum albumin (BSA) for 1 h, and plasma samples were incubated for 1 h on coated plates at dilutions of 1:250, 1:1,000, 1:4,000, and 1:16,000. Plates were then incubated with polyclonal peroxidase-conjugated antibodies directed against NHP IgG γ-chain (Sigma). Tetramethylbenzidine (TMB) (Eurobio) was finally added and optical densities measured using a Tecan plate reader (Tecan).

IFNα2 concentrations were determined using a human IFN2-specific ELISA (IFNa human matched antibody pair, Life Technologies) according to the manufacturer’s instructions, and a Tecan plate reader (Tecan).

### Luminex

The plasma concentrations of IFNγ, IL2, IL10, IL15, IL1RA, IL6, IL8, MCP1, TNFα, MIP1β and IL18 were measured using the NHP Cytokine/Chemokine Magnetic Bead Panel I (Merck). The plasma concentrations of sCD137, Granzyme B, FasL and perforin were measured using the NHP Cytokine/Chemokine Magnetic Bead Panel II (Merck). Plates were prepared according to the manufacturer’s instructions and analyzed on a Magpix instrument (Merck).

### Transcriptomic analysis

Total RNA was extracted from PBMCs using the RNeasy extraction kit (Qiagen). RNA samples quantification and qualification, mRNA poly(A)-capture and library preparations were performed as described elsewhere [23]. Sequencing was performed using a NextSeq 500 Flow Cell High Output SR75 instrument with nine samples per flow cell.

The Sequana RNA-seq pipeline was used to perform bioinformatics analyses [38]. Cutadapt version 1.11 was used to clean reads of adapter sequences and low-quality sequences [39]. Only sequences of at least 20 nucleotides in length were considered for further analysis. STAR version 2.5.0a [40], with default parameters, was used for alignment to the reference genome. Genome was downloaded from UCSC and annotation track (.gtf Ensembl Genes) was retrieved from UCSC (genome: Rhesus, assembly Nov. 2015 Mmul_8.0.1). Genes were counted using featureCounts version 1.4.6-p3 [41] from the Subreads package (parameters: -t gene -g ID -O -s 2). R software version 4.0.4 was used for statistical analyses. Insufficiently expressed genes were filtered out by removing those with no reads in at least 90% of samples and a cpm <1 for at least three samples, as recommended in the DESeq2 vignette [42]. Differential analysis was performed using the DESeq2 package [42] with a statistical model adjusted for the effect of the day, viral strain, interaction between these two factors, and pairing within monkeys. A Wald test was used for comparisons among groups and *p*-values were adjusted using the Benjamini-Hochberg multiple testing correction.

### Histology

At the terminal endpoint of the infections, the monkeys were euthanized, necropsies carried out, and the organs of interest harvested. The organ samples were fixed and inactivated in 10% formalin for a minimum of 14 days, dehydrated and immersed in paraffin using a STP120 device (Microm Microtech), and paraffin-embedded using a TES99 station (Tech-Inter). Three-micrometer thin sections were prepared using a RM2125 RTS microtome (Leica), and left to dry at room temperature. The sections were then deparaffinized with successive baths of xylene and absolute ethanol and dried before proceeding to the following steps (HE coloration or antigen immunostaining).

For hematoxylin-eosin coloration, sections were successively immersed in baths containing Gill’s hematoxylin (Sigma), lithium carbonate, and eosin (Sigma). They were then dehydrated in absolute ethanol and xylene and mounted using Eurokit glue. Images were captured using a Leica DMIL microscope with LASX software.

For immunohistochemistry, sections were processed for antigen retrieval using a Retriever 2100 with citrate buffer (pH = 6), saturated with PBS/3% BSA for 30 min, and incubated overnight in primary antibodies diluted in PBS-3% BSA at 4°C The following primary antibodies were used for immunostaining: a custom anti-Lassa mouse antibody directed against GP2c (kindly provided by the Special Pathogen Branch, CDC, Atlanta) at a 1:50 dilution, an S100A9 rabbit antibody (Invitrogen) at a 1:200 dilution, a Granzyme B mouse antibody (Abcam) at a 1:500 dilution, and an Iba-1 mouse antibody (Abcam) at a 1:500 dilution. The following day, endogenous peroxidases were quenched with 0.3% H_2_O_2_ and the sections were incubated for 30 min with a ready-to-use secondary antibody coupled with horseradish peroxidase (*N*-Histofine, Microm Microtech). The sections were then incubated with either diaminobenzidine (DAB Quanto, ThermoFisher) or 3-amino-9-ethylcarbazole (AEC, Sigma) for a length of time customized for each primary antibody, counterstained with Gill’s hematoxylin diluted 1:2 in water, briefly bathed in an ammonium hydroxide solution, dehydrated with successive baths of absolute ethanol and xylene, and mounted using xylene glue (Eukitt). Images were captured using a Leica DMIL microscope with LASX software.

## Supplementary Material

Supplemental Material

## Data Availability

The data that support the findings of this study are available from the corresponding author upon reasonable request (sylvain.baize@pasteur.fr).
