# Supplementary figures and images for: Pathogenesis of recent Lassa virus isolates from lineages II and VII in cynomolgus monkeys
Source: Virulence. 2022 Apr 18;13(1):654–69. doi: 10.1080/21505594.2022.2060170 (PMC9037461; doi:10.1080/21505594.2022.2060170)

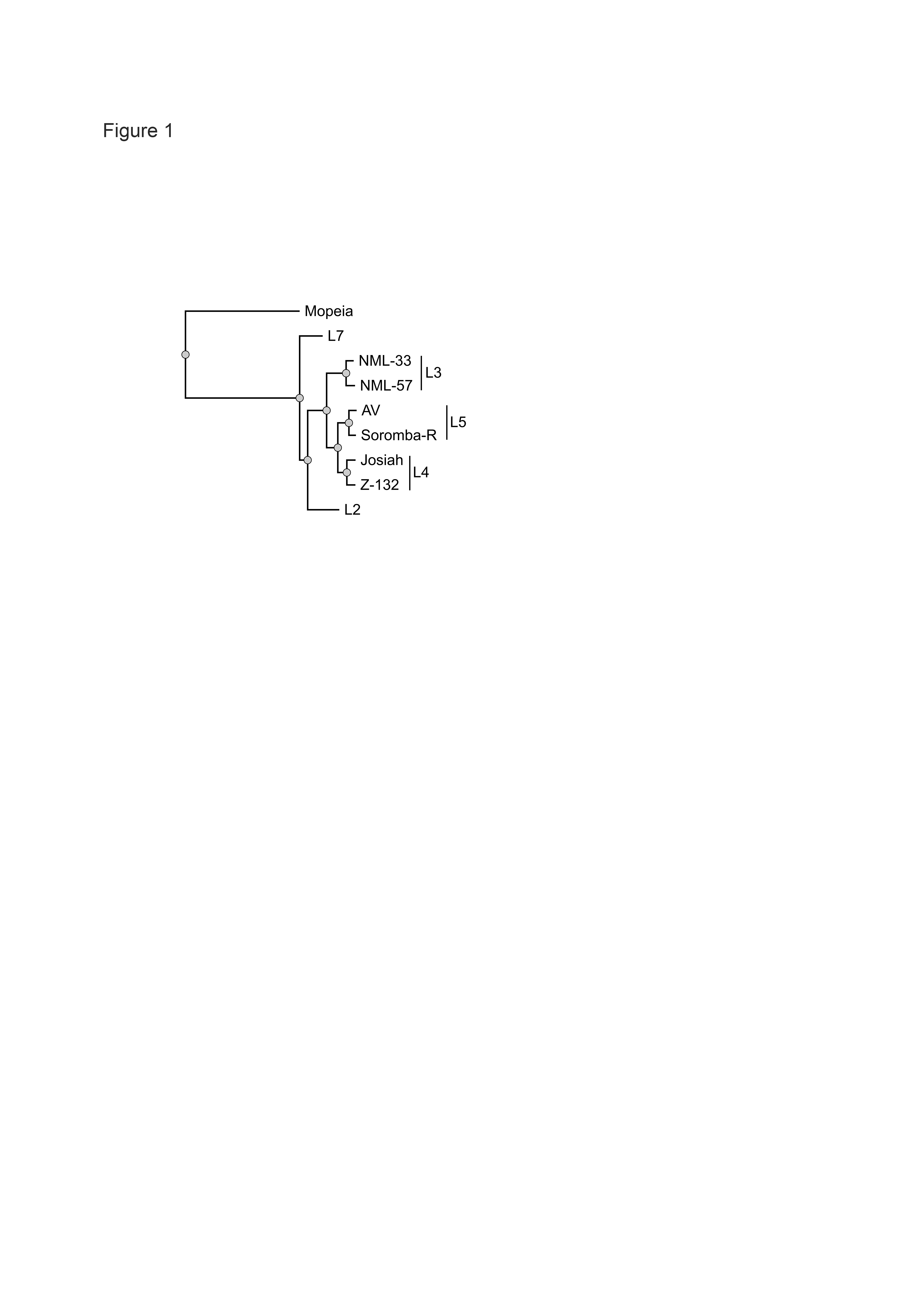

Supplement: Supplemental Material [file KVIR_A_2060170_SM2827.zip › supplementary/Suppl Fig 1.tif]

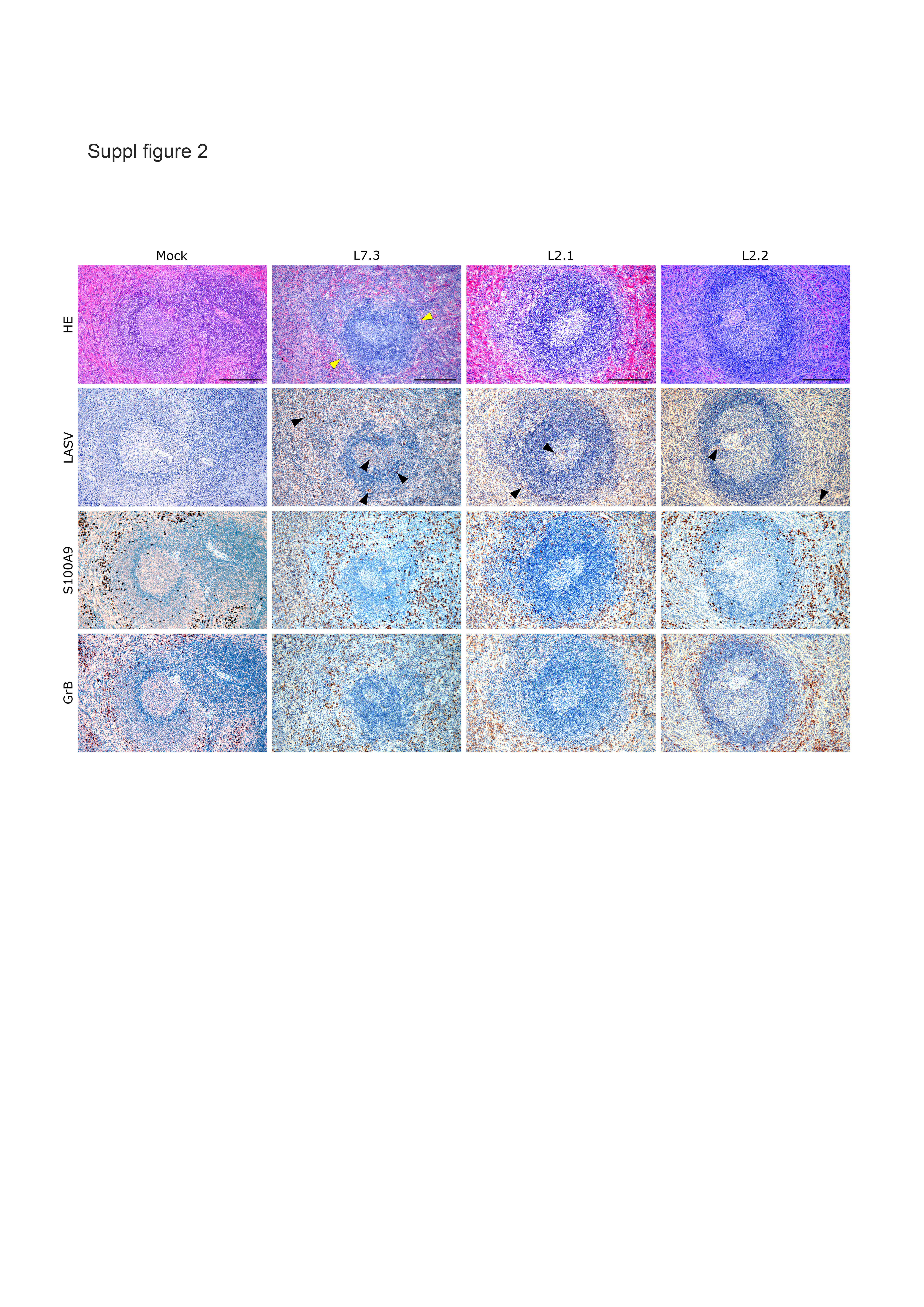

Supplement: Supplemental Material [file KVIR_A_2060170_SM2827.zip › supplementary/Suppl figure 2.tif]

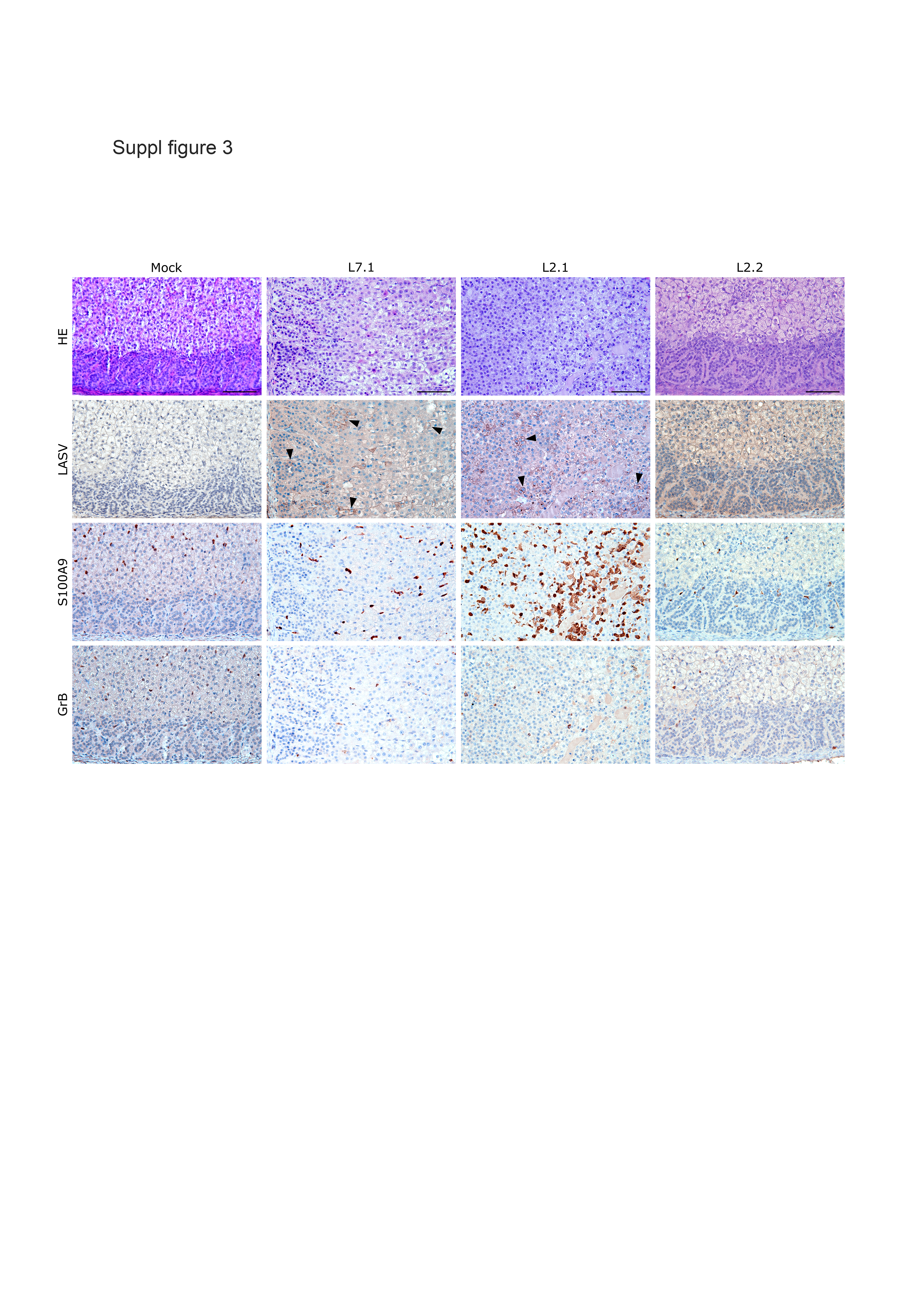

Supplement: Supplemental Material [file KVIR_A_2060170_SM2827.zip › supplementary/Suppl figure 3.tif]
